# The Role of MicroRNAs in Therapeutic Resistance of Malignant Primary Brain Tumors

**DOI:** 10.3389/fcell.2021.740303

**Published:** 2021-10-07

**Authors:** Ilgiz Gareev, Ozal Beylerli, Yanchao Liang, Huang Xiang, Chunyang Liu, Xun Xu, Chao Yuan, Aamir Ahmad, Guang Yang

**Affiliations:** ^1^Central Research Laboratory, Bashkir State Medical University, Ufa, Russia; ^2^Department of Neurosurgery, The First Affiliated Hospital of Harbin Medical University, Harbin, China; ^3^Institute of Brain Science, Harbin Medical University, Harbin, China; ^4^Interim Translational Research Institute, Academic Health System, Hamad Medical Corporation, Doha, Qatar

**Keywords:** malignant primary brain tumors, miRNAs, resistance, therapy, oncogenesis, exosomes

## Abstract

Brain tumors in children and adults are challenging tumors to treat. Malignant primary brain tumors (MPBTs) such as glioblastoma have very poor outcomes, emphasizing the need to better understand their pathogenesis. Developing novel strategies to slow down or even stop the growth of brain tumors remains one of the major clinical challenges. Modern treatment strategies for MPBTs are based on open surgery, chemotherapy, and radiation therapy. However, none of these treatments, alone or in combination, are considered effective in controlling tumor progression. MicroRNAs (miRNAs) are 18–22 nucleotide long endogenous non-coding RNAs that regulate gene expression at the post-transcriptional level by interacting with 3′-untranslated regions (3′-UTR) of mRNA-targets. It has been proven that miRNAs play a significant role in various biological processes, including the cell cycle, apoptosis, proliferation, differentiation, etc. Over the last decade, there has been an emergence of a large number of studies devoted to the role of miRNAs in the oncogenesis of brain tumors and the development of resistance to radio- and chemotherapy. Wherein, among the variety of molecules secreted by tumor cells into the external environment, extracellular vesicles (EVs) (exosomes and microvesicles) play a special role. Various elements were found in the EVs, including miRNAs, which can be transported as part of these EVs both between neighboring cells and between remotely located cells of different tissues using biological fluids. Some of these miRNAs in EVs can contribute to the development of resistance to radio- and chemotherapy in MPBTs, including multidrug resistance (MDR). This comprehensive review examines the role of miRNAs in the resistance of MPBTs (e.g., high-grade meningiomas, medulloblastoma (MB), pituitary adenomas (PAs) with aggressive behavior, and glioblastoma) to chemoradiotherapy and pharmacological treatment. It is believed that miRNAs are future therapeutic targets in MPBTs and such the role of miRNAs needs to be critically evaluated to focus on solving the problems of resistance to therapy this kind of human tumors.

## Introduction

Malignant primary brain tumors (MPBTs) are one of the most difficult to treat types of tumors, resulting in significant morbidity and mortality in both children and adults. The most common MPBTs are glioblastomas, high-grade meningiomas, medulloblastoma (MB), and pituitary adenomas (PAs) with aggressive behavior, as aggressive prolactin PAs (Schiff and Alyahya, 2020; Thakkar et al., 2021). For example, for patients with glioblastoma or MB, the overall survival remains is poor, with conventional therapies such as radio- and chemotherapy only providing marginal benefits to patient survival. Therefore, new strategies are needed to overcome the barriers to successful treatment (Thakkar et al., 2021).

**FIGURE 1:**
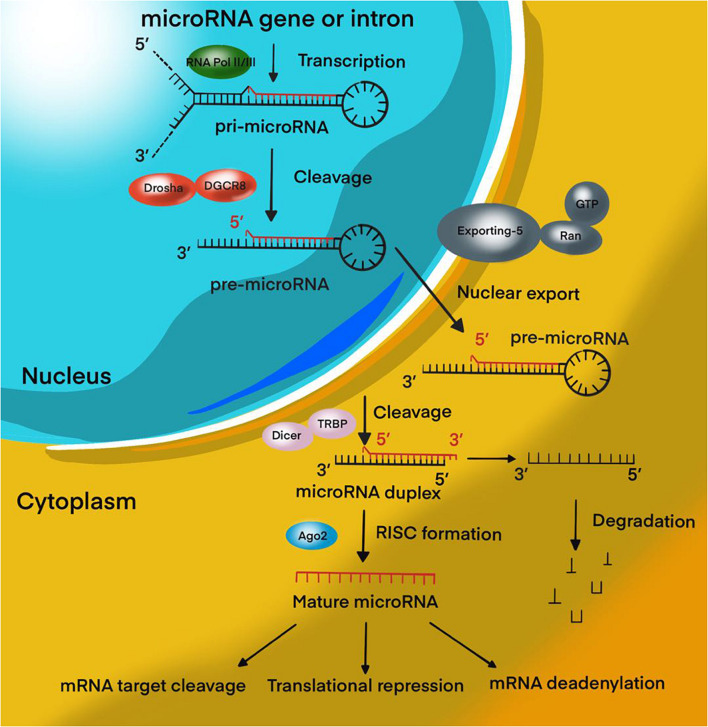
MiRNA biogenesis pathway. Overview schematic representation of canonical miRNA biogenesis pathway.

Over the past decades, significant progress has been achieved in the study of tumor biology, the study of the mechanisms of control of tumor metastasis, apoptosis, invasion, angiogenesis, and proliferation of tumor cells. These data were obtained by studying the cellular composition and microenvironment of tumors, various intracellular signaling pathways, and molecular processes of oncogenesis (Richardson et al., 2020). MicroRNAs (miRNAs) are 18–22 nucleotide endogenous non-coding RNAs that regulate gene expression at the post-transcriptional level by interacting with 3′-untranslated regions (3′-UTR) of mRNA-targets (Lu and Rothenberg, 2018; Figure 1). It is estimated that more than 60% of all human protein-coding genes are directly regulated by miRNAs (Ha and Kim, 2014; Dexheimer and Cochella, 2020). It has been proven that miRNAs are involved in various biological processes, including the cell cycle, apoptosis, cell proliferation, and differentiation (Rupaimoole and Slack, 2017; Saliminejad et al., 2019). In addition, miRNAs play a role in the oncogenesis of various human tumors, including brain tumors (Bertoli et al., 2015; Qadir and Faheem, 2017; Ali Syeda et al., 2020; Balachandran et al., 2020). Recently, most research has focused on the role of miRNAs in resistance to malignant human tumors therapy. In MPBTs, the role of miRNAs in radio- and chemotherapy resistance is an attractive area of research and is expected to lead to the development of novel treatment strategies. This review will focus on differential expression of miRNAs in MPBTs (e.g., high-grade meningiomas, MB, PAs with aggressive behavior, and glioblastoma) with their gene-targets and their potential role in resistance to radio- and chemotherapy, and to pharmacological treatment.

## Micrornas Dysregulation in Malignant Primary Brain Tumors

MicroRNAs perform an important function in the complex mechanism of regulation of gene activity, since they determine the qualitative and quantitative composition of transcripts and proteins necessary for the development of individual tissues, organs and the whole organism. A growing body of evidence points to the importance of miRNAs deregulation in the initiation and progression of tumors, where they can act as oncogenic miRNAs (oncomiRs) or tumor-suppressor miRNAs, depending on the cellular function of their gene-targets (Liu et al., 2014). Moreover, the activation or suppression of specific miRNA families is the mechanism by which oncogenes such as epidermal growth factor receptor (*EGFR*) and *MET* or tumor suppressor genes such as phosphatase and tensin homolog deleted on chromosome 10 (*PTEN*), adenomatous polyposis coli (*APC*), and breast cancer type 1/2 (*BRCA1/2*) induce or inhibit oncogenesis (Zhang et al., 2007; Lee and Muller, 2010).

To date, a lot of evidence has been collected about the aberrant expression of miRNAs in various tumors, particular, malignant (Van Roosbroeck and Calin, 2017). It was shown that miRNAs control the expression of genes of regulatory pathways that play a key role in tumor development, control apoptosis and proliferation of tumor cells, tumor growth in response to DNA damage and repair, angiogenesis and response to hypoxia, the interaction of tumor cells with the microenvironment (Rupaimoole and Slack, 2017; Van Roosbroeck and Calin, 2017). A number of researchers isolate miRNAs directly related to individual processes in a tumor, or stages of development, of a disease (Vishnoi and Rani, 2017). Many miRNAs are involved in key signaling pathways associated with the regulation of the cell cycle and apoptosis. In one of the latest studies, Allahverdi et al. (2020) found that adipose-derived mesenchymal stem cells (AD-MSCs) delivering miR-4731 induces apoptosis and cell cycle arrest in the glioblastoma cell line. Another study provided evidence that miR-221-3p reduces MB cell proliferation by inducing apoptosis and G0/G1 arrest by suppressing eukaryotic translation initiation factor 5A-2 (*EIF5A2*) (Yang et al., 2019).

There is also a separate group of miRNAs associated with the metastatic activity of tumors – metastamiRs. Moreover, among such miRNAs, some promote (miR-9, miR-210, miR-21, miR-218, etc.) tumor metastasis, while others (miR-145, miR-7, miR-146-a, etc.), on the contrary, suppress it (Alsidawi et al., 2014; Lu et al., 2015; Lima et al., 2017; Ji et al., 2018; Maryam et al., 2021).

In recent years, thanks to advances in molecular oncology, it has been possible to decipher some of the mechanisms of oncogenesis and to determine the signs of a malignant phenotype, one of which is angiogenesis. Malignant tumors requires more oxygen and nutrients to grow (Viallard and Larrivée, 2017). The solution to this issue is to trigger the mechanism of angiogenesis in the tumor. Vascular endothelial growth factor (*VEGF*) is extremely important for the formation of an adequate functioning vascular system during embryogenesis and in the early postnatal period, but it also plays an important role in pathological angiogenesis. In many types of tumors, increased *VEGF* expression correlates with poor prognosis, including aggressive tumor growth, recurrence, metastasis, and decreased survival (Melincovici et al., 2018). In addition, *VEGF* expression correlates with a decrease in the density of the microvascular network in the malignant brain tumors, which in itself serves as an indicator of the prognosis of vascular rupture, followed by hemorrhage in the tumor bed (Apte et al., 2019). To date, a number of miRNAs have been identified that are highly expressed in endothelial cells (ECs) and/or are activated under hypoxic conditions. Among these miRNAs, it is worth noting miR-126, which is specifically expressed in the ECs and is a key regulator of the integrity of the vascular wall and angiogenesis in various tumors, including brain tumors (Fish et al., 2008). Smits et al. (2012) showed there is significant low-expression of miR-125b in ECs co-cultured with U87 glioblastoma line cells. Moreover, the authors demonstrated that miRNA-125b inhibits angiogenic processes by directly regulating *Myc*-associated zinc finger protein (*MAZ*)/*VEGF* signaling pathway expression. It is known that, *MAZ*-binding sites are located in the promoter regions of angiogenic factor *VEGF*.

Xiao et al. (2016) demonstrated that miR-566 was overexpressed in glioblastoma *in vitro* and *in vivo*, and inhibition of miR-566 was able to suppress the invasion and migration of glioblastoma cells, and angiogenesis via the *VEGF*/Von Hippel–Lindau tumor suppressor (*VHL*) pathway. This suggests that miR-566 may function as an oncogene, and therefore, miR-566 may be considered a novel therapeutic target of glioblastoma (Xiao et al., 2016).

There is evidence that some miRNAs can participate in the processes of malignant transformation in benign brain tumors. Brain tumors of different histology are characterized by specific miRNAs expression profiles associated with the clinical and pathological properties of the tumor (Van Roosbroeck and Calin, 2017). For instance, miR-21 makes it possible to distinguish between the main histological types of meningioma, for which miR-21 expression showed a significant increase in World Health Organization (WHO) grade 2 and 3 lesions as compared to WHO grade 1 lesions (Katar et al., 2017).

Currently, there is an active search for new miRNAs and their target genes involved in other important processes associated with oncogenesis (and not only), for example, the control of the balance of self-renewal and differentiation of stem cells, epithelial-mesenchymal transition (EMT), regulation of the immune response, the relationship of the tumor with the microenvironment, etc.

It should also be borne in mind that the reason for the change in the expression of miRNA may be a violation of the expression of proteins involved in miRNA biogenesis. It is known that the loss or insufficient expression of Drosha, Dicer, and TRBP can lead to the development of a tumor process (Olejniczak et al., 2018). Several groups showed impaired expression of Drosha and Dicer in glioblastoma, pineoblastoma, and neuroblastoma, all of which correlated with a poor prognosis of survival (Lin et al., 2010; Mansouri et al., 2016; de Kock et al., 2020). Decreased expression of these proteins can be mediated by mutations or epigenetic inactivation of their genes. In addition, mutations in Dicer can lead to impaired recognition of miRNA precursors and a change in the balance of strands. Argonaute 2 (Ago2) and GW proteins that act as direct partners of miRNAs are often susceptible to somatic mutations in glioblastoma, which are accompanied by a high level of instability of microsatellite tumor DNA (Li S. et al., 2014; Li Z. et al., 2019). Disruption of the transport of pre-miRNAs into the cytoplasm can also lead to a decrease in their expression. A mutation in the exportin-5 gene, leading to the synthesis of a truncated protein that is unable to recognize pre-miRNAs, causes a decrease in the level of mature miRNAs in a number of tumors (Wu et al., 2018).

A number of oncogenic proteins can directly interfere with miRNA biogenesis in tumors. Thus, wild and mutant forms of p53 are involved in the biogenesis of a number of miRNAs, primarily miR-34 (Zhang et al., 2019). It is known that mutant forms of p53 can inhibit Drosha activity and prevent the formation of pre-miRNA (Garibaldi et al., 2016). Transforming growth factor beta (*TGF-*β) affects the processing of miRNA through the binding of effector proteins to the microprocessor complex and pri-miRNA. YES-associated protein 1 (*YAP1*), one of the components of the Hippo pathway, regulates the activity of the microprocessor complex depending on the density of cells in culture (Ruan et al., 2016). Tumor suppressor protein *BRCA1* also stimulates the activity of the microprocessor complex (Lee and Muller, 2010). Under hypoxic conditions, activated *EGFR* can phosphorylate Ago2, inhibiting its interaction with Dicer and decreasing the level of activity of miRNA effector systems (Shen et al., 2013). In addition, the interaction of p53 with Ago2 leads to a change in the spectrum of miRNAs associated with it, leading to the formation of complexes carrying tumor suppressor miRNAs, for example, let-7 (Krell et al., 2016).

Thus, miRNAs are one of the key factors in the development of malignant forms of brain tumors, both as drivers of malignant transformation and as a victim of the deregulation of cellular regulatory systems. The close relationship of miRNAs with MPBTs has led to the fact that they are currently being actively studied (Table 1; Grunder et al., 2011; Kliese et al., 2013; Asuthkar et al., 2014; Wang et al., 2015; Wei et al., 2015; Yu et al., 2016; Zheng et al., 2017; Xu et al., 2018; Xue et al., 2019; Hou et al., 2020; Muñoz-Hidalgo et al., 2020; Negroni et al., 2020; Song et al., 2020), including with the aim of creating diagnostic and therapeutic systems designed to increase the effectiveness of treatment of this disease.

**TABLE 1:** The most relevant studies to study the role of miRNAs in the oncogenesis of malignant primary brain tumors (MPBTs).

Tumor type	miRNA	Gene-target	Biological function	Regulation	Phenotype	References
Glioblastoma	miR-191	*NDST1*	Promote tumor growth and cells migration	Up	OncomiR	Xue et al., 2019
Glioblastoma	miR-200c	*ZEB1*	Inhibit tumor growth and cells migration	Down	Tumor suppressor	Muñoz-Hidalgo et al., 2020
Glioblastoma	miR-449b-5p	*WNT2B/Wnt/*β*-*catenin	Inhibits tumor cells proliferation, invasion, and migration	Down	Tumor suppressor	Hou et al., 2020
Atypical and anaplastic meningiomas	miR-145	*COL5A1*	Inhibit motility and proliferation of tumor cells	Down	Tumor suppressor	Kliese et al., 2013
Anaplastic meningioma	miR-195	*FASN*	Inhibit proliferation, migration, and invasion	Down	Tumor suppressor	Song et al., 2020
Atypical and anaplastic meningiomas	miR-497∼195 cluster	*GATA-4*	Decreases tumor cell viability	Down	Tumor suppressor	Negroni et al., 2020
Atypical and anaplastic meningiomas	miR-224	*ERG2*	Promote tumor growth and reduce apoptosis of cells. Associated with poor prognosis	Up	OncomiR	Wang et al., 2015
MB	miR-21	*PDCD4*	Decrease the motility of tumor cells and reduce their migration	Up	OncomiR	Grunder et al., 2011
MB	miR-211	*PI3K/AKT* and *mTOR*	Inhibit growth, migration and invasion	Down	Tumor suppressor	Xu et al., 2018
MB	miR-494	*MMP-9* and *SDC1*	Reduce tumor growth and angiogenesis	Down	Tumor suppressor	Asuthkar et al., 2014
Invasive PA (NFA, GH, ACTH, PRL)	miR-106b	*PTEN-PI3K/AKT/MMP-9*	In tumor cells induces invasive properties	Up	OncomiR	Zheng et al., 2017
Invasive PA (NFA, GH, ACTH, PRL)	miR-26a	*PLAG1*	In tumor cells induces invasive properties. Associated with poor prognosis	Up	OncomiR	Yu et al., 2016
Pituitary carcinoma	miR-20a, miR-106b and miR-17-5p	*PTEN* and *TIMP2*	Activation of invasive properties and migration in a tumor cells. Carcinoma metastasis	Up	OncomiR	Wei et al., 2015

*miR, microRNA; MB, medulloblastoma; PA, pituitary adenoma; miR, microRNA; NFA, non-functioning adenoma; GH, growth hormone-secreting adenoma; FSH, follicle-stimulating hormone adenoma; LH, luteinizing hormone-secreting adenoma; ACTH, adrenocorticotropic hormone-secreting adenoma; PRL, prolactin-secreting adenoma; *NDST1*, Bifunctional heparan sulfate N-deacetylase/N-sulfotransferase 1; *ZEB1*, E-box-binding homeobox 1; *WNT2B*, Wnt family member 2B; *COL5A1*, collagen type V alpha 1 chain; *FASN*, fatty acid synthase; *GATA-4*, GATA binding protein 4; Early growth response 2; *MMP-9*, matrix metallopeptidase 9; *PDCD4*, programmed cell death 4; *AKT*, protein kinase B; *mTOR*, mammalian target of rapamycin; *SDC1*, Syndecan 1; *PTEN*, tensin homolog deleted on chromosome 10; *PI3K*, phosphoinositide 3-kinases; *PLAG1*, pleomorphic adenoma gene 1; *TIMP2*, tissue inhibitor of metalloproteinases 2.*

## Micrornas in Resistance to Chemoradiotherapy and Pharmacological Treatment

**FIGURE 2:**
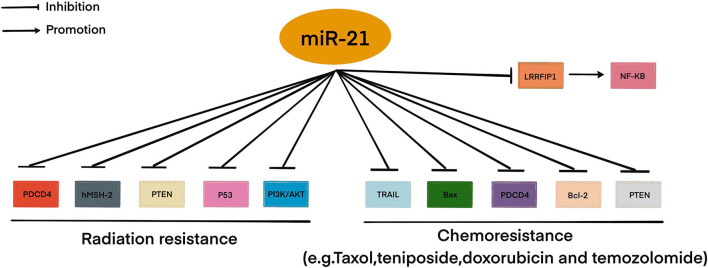
MiR-21 involved in radio- and chemoresistance in glioblastoma. This figure shows through which signaling pathways miR-21 may be involved in resistance to therapy in glioblastoma.

Resistance to therapy of brain tumors is an important problem in modern neurosurgery. There are two main mechanisms for the emergence of radio- and chemotherapy resistance: (1) activation of specific signaling pathways responsible for the “neutralization” of the chemotherapy drug and ionizing radiation in the tumor cell. Such these signaling pathways include phosphoinositide 3-kinases/protein kinase B (*PI3K/AKT*) è mitogen-activated protein kinase (*MAPK*)/*ERK*; and 2) violation of the mechanism of cell death under the influence of chemotherapy and ionizing radiation (Cao et al., 2019; Kapoor et al., 2020). This mechanism includes the blocking of apoptosis with p53 mutations, overexpression of B-cell lymphoma 2 (*Bcl-2*), a decrease in the expression of cluster of differentiation 95 (CD95) (Kapoor et al., 2020). It is now known that miRNAs can control the regulation of target genes or signaling pathways involved in malignant tumor resistance to therapy, including MPBTs. Among these miRNAs, miR-21 is the most studied in glioblastoma. MiR-21 is one of the important miRNAs involved in glioblastoma oncogenesis. A large number of studies indicated that miR-21 could affect a variety of cellular and molecular pathways. It has been showing that deregulation of miR-21 could be associated with resistance to radio- and chemotherapy of glioblastoma (Figure 2; Lan et al., 2015; Masoudi et al., 2018; Buruiană et al., 2020). In this article, we will consider current knowledge about the role of miRNAs in the mechanisms of resistance to therapy in MPBTs. In addition, a summary of the role of some miRNAs in therapy resistance by targeting their gene targets is shown in Table 2 (Pannuru et al., 2014; Dénes et al., 2015; Abdelfattah et al., 2018; Chen et al., 2018; Li J. et al., 2019; Bogner et al., 2020; Hu et al., 2020; Sun et al., 2020; Zhao C. et al., 2020; Cardoso et al., 2021) and Table 3 (Lee et al., 2014; Li W. et al., 2014; Huynh et al., 2015; Wu et al., 2015; Yang F. et al., 2017; Zhang Q. et al., 2020).

**TABLE 2:** MiRNAs involved in the regulation of chemotherapeutic and pharmacological drug treatment resistance in malignant primary brain tumors (MPBTs).

miRNA	Type of tumor	Type of drug	Drug	Gene-target	Mechanism	References
miR-128-3p	Glioblastoma	DNA-targeted drugs	TMZ	*c-Met/EMT* (*c-Met, PDGFR*α, *Notch1*, and *Slug*)	Reduces the proliferation, invasion, and migration	Zhao C. et al., 2020
miR-186	Glioblastoma	DNA-targeted drugs	Cisplatin	*YY1*	Inhibits the formation of the GIC phenotype	Li J. et al., 2019
miR-302a	Glioblastoma	Tyrosine kinase inhibitors	Cediranib	*PGK1*	Decrease glycolysis, cell growth, migration, and invasion	Cardoso et al., 2021
miR-let-7f-1	MB	DNA-targeted drugs	Cisplatin	*HMGB1*	Inhibit autophagy	Pannuru et al., 2014
miR-29c-3p	MB	DNA-targeted drugs	Cisplatin	*Bcl-2/Wnt2*	Increase apoptosis	Sun et al., 2020
miR-584-5p	MB	Tubulin inhibitors	Vincristine	*HDAC1/eIF4E3*	Cause cell cycle arrest, DNA damage, and spindle defects	Abdelfattah et al., 2018
miR-31	MB	DNA-targeted drugs	Ginsenoside Rh2	*Wnt/*β*-catenin*	Inhibit the proliferation and migration, and induce apoptosis	Chen et al., 2018
miR-197	Meningioma	DNA-targeted drugs	Quercetin	*Bcl-2/Bax*	Reduce tumor cell proliferation and increase apoptosis	Hu et al., 2020
miR-34a	Aggressive somatotropinoma	SSAs	Octreotide	*cAMP*	Antiproliferative effect	Bogner et al., 2020
miR-1299	Aggressive prolactinoma	DA	Bromocriptine	*FOXO1*	Promotes the synthesis and secretion of prolactin	Dénes et al., 2015

*miR, MicroRNA; MB, medulloblastoma; SSAs, somatostatin analogs; DA, dopamine antagonist; TMZ, Temozolomide; EMT, epithelial-mesenchymal transition; *PDGFR*α, platelet-derived growth factor receptor A; *YY1*, Yin Yang 1; *PGK1*, phosphoglycerate kinase 1; *HMGB1*, high-mobility group protein B1; *Bcl-2*, B-cell lymphoma-2; *Wnt2*, wingless-type MMTV integration site family, member 2; *HDAC1*, histone deacetylase 1; *IF4E3*, eukaryotic translation initiation factor 4E type 3.*

**TABLE 3:** Role miRNAs in regulating malignant primary brain tumors (MPBTs) radiosensitivity.

miRNA	Type of tumor	Gene-target	Regulation	Mechanism	Response	References
miR-221/222	Glioblastoma	*Akt*	Down	Inhibit tumor growth	Increase radiosensitive	Li W. et al., 2014
miR-181d	Glioblastoma	*NF-*κ*B*	Up	Suppress tumor cell proliferation, colony formation and anchor-independent growth, as well as migration, invasion and tube formation	Increase radiosensitive	Yang F. et al., 2017
miR-124-3p	Glioblastoma	*mTOR, MAPK, TGFbeta*, and *PI3K-Akt*	Up	Inhibit tumor growth and promote apoptosis	Increase radiosensitive	Wu et al., 2015
miR-205	Glioblastoma	*GRP78, c-Myc*,β*-catenin* and *vimentin*	Up	Decreases tumor-sphere-formation and colony-forming abilities, inhibit migration and invasion	Increase radiosensitive	Huynh et al., 2015
miR142-3p	MB	*Sox2* and *ADCY9*	Down	Elevate the expression of miRNA decreases cancer stem-like characteristics and stemness	Increase radioresistance	Lee et al., 2014
miR-584-5p	MB	*HDAC1/eIF4E3*	Up	Cause cell cycle arrest, DNA damage, and spindle defects	Increase radiosensitive	Abdelfattah et al., 2018
miR-221/222	Meningioma	*PTEN*	Down	Inhibit tumor cell proliferation, invasive and colony formation, and promote apoptosis	Increase radiosensitive	Zhang Q. et al., 2020

*miR, MicroRNA; MB, medulloblastoma; *Akt*, protein kinase B alpha; *NF-*κB, nuclear factor kappa-light-chain-enhancer of activated B cells; *mTOR*, mammalian target of rapamycin; *MAPK*, mitogen-activated protein kinase; *TGF-beta*, transforming growth factor beta; *PI3K*, phosphoinositide 3-kinases; *GRP78*, glucose regulatory protein 78; *Sox2*, SRY-Box transcription factor 2; *ADCY9*, adenylate cyclase 9; *HDAC1*, histone deacetylase 1; *eIF4E3*, eukaryotic translation initiation factor 4E family member 3; *PTEN*, phosphatase and tensin homolog deleted on chromosome 10.*

### Glioblastoma

Glioblastoma is the most aggressive primary brain tumor and usually has a poor prognosis. Thus, the median survival rate of patients with glioblastoma after surgical resection and standard radio- and chemotherapy is no more than 12–15 months, while the 2-year survival rate for this group of patients varies from 26 to 33% (Wirsching et al., 2016; Schiff and Alyahya, 2020). Angiogenesis is the most important pathophysiological mechanism for the growth and progression of glioblastoma due to the active development of the microvascular network. The accelerated development of the microvascular network in glioblastoma occurs due to the synthesis of a large number of growth factors by tumor cells, including the *VEGF* family, placental growth factor (*PLGF*), platelet-derived growth factor (*PDGF*), and fibroblast growth factor (*FGF*) (Le Rhun et al., 2019). It should be noted that the microvascular network of glioblastoma is characterized by a high degree of tortuosity, increased permeability, as well as an increased diameter of the vascular lumen, and a thickened basement membrane (Ahir et al., 2020). It is believed that these features of the microvascular network of glioblastoma increase the hypoxia of the tumor tissue, thereby reducing the effectiveness of the use of cytotoxic drugs. It is for this reason that the development and use of anti-angiogenic drugs seem to be one of the most promising methods of targeted treatment of glioblastoma patients. The effectiveness of the use of anti-angiogenic drugs in the treatment of glioblastoma has been clearly demonstrated in a number of clinical studies (Sousa et al., 2019; Schulte et al., 2020). However, the widespread use of anti-angiogenic drugs in clinical practice has led to the development of glioblastoma resistance to drugs of this group. The formation of drug resistance of glioblastoma to anti-angiogenic drugs is associated with molecular and cellular features of the behavior of tumor cells, including with the participation of certain miRNAs (Zeng et al., 2018b).

Autophagy is one of the main mechanisms of tumor resistance. It should be noted that autophagy is the main cellular defense mechanism in the development of hypoxic conditions, which does not require tissue and extracellular matrix remodeling (Kimmelman and White, 2017). As is already known, the use combination of anti-angiogenic therapy with radiotherapy leads to the development of tumor tissue hypoxia due to the disturbance of the angiogenesis process necessary for the growth and progression of the tumor (Le Rhun et al., 2019). It is generally accepted that the process of autophagy in tumor cells is aimed at the destruction of proteins and signaling molecules formed during hypoxic conditions, which leads to the preservation of intracellular structures and the leveling of the secondary effects of anti-angiogenic drugs. It is assumed that in glioblastoma, drug resistance to bevacizumab is associated with non-selective hypoxia-induced factor (*HIF*)-dependent autophagy-mediated through the activity of the protein-interacting protein 3 (*BNIP 3*) and hypoxia-inducible factor 1-alpha (*HIF1-*α) protein (Hu et al., 2012). It is known that *HIF-1*α can cause cell cycle arrest, initiating angiogenesis, and regulating cellular metabolism (Gabriely et al., 2017; Huang et al., 2019). Huang et al. identified that the expression of *HIF-1*α was increased in glioblastoma *in vitro* and *in vivo* under hypoxia (Cardoso et al., 2021). Moreover, the longer the duration of hypoxia, the higher was the expression of *HIF-1*α. However, the expression of miR-224-3p was decreased under hypoxia conditions in a time-dependent manner. Their data showed that the miR-224-3p mimic significantly suppressed the expression *HIF-1*α and inhibited cell mobility while increased chemosensitivity to Temozolomide (TMZ) of glioblastoma. In addition, the miR-224-3p mimic suppressed the expression of *VEGF* with an increased cell apoptosis rate. In another study, miR-203 could be a useful target for overcoming the radioresistance of glioblastoma by suppressing *HIF-1*α expression *in vitro* (Chang et al., 2016). However, further experiments are needed to understand the complex link between miR-203 and *HIF-1*α expression.

### Medulloblastoma

Medulloblastoma is a malignant primary tumor of the posterior fossa (WHO grade 4), mainly manifested in children. MB arises in the posterior fossa, usually from the cerebellar vermis and in the roof of the fourth ventricle (Schiff and Alyahya, 2020). MBs tend to metastasize along with the cerebrospinal fluid (CSF) pathways, which is detected in 35% of cases at the time of diagnosis. MBs are the most common malignant neoplasms of the brain in childhood and account for 15 to 30% of all primary CNS tumors in children, and about 70% of all cases are diagnosed in children under 15 years of age (Quinlan and Rizzolo, 2017). The age peak of diagnosis is between 3 and 5 years, and only 25% are patients between the ages of 20–44 (Millard and De Braganca, 2016; Quinlan and Rizzolo, 2017). Patients with MB have a poor outcome despite surgical, radio- and chemotherapy. However, the molecular mechanisms that confer sensitivity or resistance of MB to chemoradiation therapy are still unclear.

Recent evidence has implicated miRNAs in modulating chemo- and radiosensitivity in MBs (Joshi et al., 2019). It has been shown that in MB therapy there is a balance between cell cycle arrest and cell death (Kasuga et al., 2008). Melanoma-associated antigen-A (*MAGE-A*) family acts as a cell cycle regulatory protein and plays a key role in the oncogenesis and therapy resistance in MB. Kasuga et al. (2008) demonstrate that knockdown of *MAGE-A* increases apoptosis and sensitizes MB cells to chemotherapeutic agents such as cisplatin and etoposide. This finding supports the hypothesis that knockdown of *MAGE-A* genes increases the susceptibility of MB cells to cisplatin and etoposide, potentially by accumulating cells in the S phase. In contrast, Sheamal et al. showed that miR-34a directly targets the *MAGE-A* family (*MAGE-A2, MAGE-A3, MAGE-A6*, and *MAGE-A12*), disengaging p53 from *MAGE-A*–mediated repression (Weeraratne et al., 2011). Moreover, an important consequence of this is a positive feedback loop that sensitizes MB cells to cisplatin and etoposide via delayed G2/M progression and increased tumor cells apoptosis.

There is evidence that phosphatase and tensin homolog deleted on chromosome 10 (*PTEN*) dysfunction plays a crucial role in the development and progression MB. *PTEN* plays important roles in many cellular processes, including cell-cycle progression and apoptosis (Tolonen et al., 2020). Li et al. (2015) reported the upregulation of miR-106b in MB. In their study, the suppression of miR-106b inhibited cell proliferation, migration and invasion, and anchorage-independent growth, tumorsphere formation. In addition, downregulation of miR-106b suppressed the tumor growth by promoting G1 arrest and apoptosis. Besides, *PTEN* can be modulated by miR-106 family in various human cancers. For instance, miR-106b caused cell radio resistance in colorectal cancer via the *PTEN/PI3K/AKT* pathways (Zheng et al., 2015). MiR-106a induced cisplatin resistance via the *PTEN*/*AKT* pathway in gastric cancer cells (Fang et al., 2013). However, the role of miR-106b in therapy resistance of MB is still largely unknown. Nevertheless, this is an excellent opportunity to continue research on the role of miR-106b directly targeted *PTEN* in therapy resistance of MB.

### Pituitary Adenomas With Aggressive Behavior

Among the tumors of the chiasmatic-sellar region, the most common are PAs, accounting for about 18% of all tumors of this localization. In the overwhelming majority of cases, these are benign neoplasms, characterized by slow growth rates and progression (Schiff and Alyahya, 2020). However, among them, there are PAs with aggressive behavior, which exhibits the properties of resistance to traditional treatment methods (Lake et al., 2020). Among PAs, the first place is occupied by tumors accompanied by the syndrome of hyperprolactinemia – prolactinomas, as well as non-functioning (hormonally inactive) PAs, each approximately 40%. The next most frequent is somatotropinomas, about 13–15%, accompanied by symptoms of acromegaly. Gonadotropin-secreting PAs, ACTH-secreting PAs, GH-secreting PAs, mixed forms are less common. In the age range, PAs occupy a period from 30 to 50 years, which is the working age (Elsarrag et al., 2020; Lake et al., 2020). In connection with all of the above, PAs, their diagnosis, and especially, treatment are important medical and social problems.

The main methods of treatment for PAs are surgical removal of the tumor and pharmacological treatment and their combinations. Radio- and chemotherapy are used, as a rule, when it is impossible to perform a surgical intervention or when it is at high risk, and when the tumor is highly aggressive (Fleseriu and Popovic, 2020). Since there is no clear definition and availability of reliable prognostic markers, PAs with aggressive behavior are difficult to identify at initial presentation, and therefore the primary therapeutic approach is no different from other PAs depending on the type of tumor (Mete and Lopes, 2017). Resistance to drugs presenting as escalating hormone levels and/or tumor growth where can be an early indicator of aggressiveness. There are many studies examining changes in miRNA expression in PAs. Among them are studies on their role in drug resistance in various types of PAs (Ciato and Albani, 2020). However, there is no evidence base on their potential role in resistance to chemo- and radiotherapy in patients with PAs with aggressive behavior and pituitary carcinomas. It is possible to suggest from previous studies which miRNAs and through which signaling pathways can participate in the mechanisms of resistance to chemo- and radiotherapy. For instance, in a recent study, Wang Z. et al. (2019) successfully identified one key target gene, *EGFR*, and two crucial miRNAs, miR-489 and miR-520b, associated with aggressiveness of prolactinomas based on bioinformatics analysis. It is also known that *EGFR* is one of the most frequently altered oncogenes in tumors, which important role in therapy resistance and is often associated with a negative prognosis (Lee and Muller, 2010).

Prolactinoma is the most commonly seen secretory tumor of pituitary glands. More than 90% of prolactinomas are microprolactinomas (<1.0 cm), while the rest are macroprolactinomas (≥1.0 cm). Macroprolactinomas account for approximately half of all functioning pituitary macroadenomas (Huynh et al., 2021). Without a doubt, prolactinoma is an innocent tumor of its kind. Prolactinomas are more aggressive and are characterized by increased proliferative ability; they can turn into recurrent, invasive giant prolactinomas (Castinetti et al., 2021). Most patients with prolactinomas respond to standard doses of dopamine agonists (DA), while the rest of the patients remain resistant to therapy. In this case, the term is used – resistance to DA, the inability to achieve normalization of prolactin levels, and tumor reduction by 50%, while taking the maximum tolerated dosage of the drug (Giraldi and Ioachimescu, 2020). To overcome resistance, it is necessary to increase the dose of drugs, in this regard, the risk of developing side effects, such as liquorrhea, headaches, acute psychosis, etc., (Molitch, 2020; Vermeulen et al., 2020; Castinetti et al., 2021). Surgical intervention is indicated in case of intolerance or resistance to DA, or with a persistent increase in tumor size with the development of neuro-ophthalmic symptoms, or if there is a rapid loss of vision or cranial nerve paralysis due to intratumoral hemorrhage (Panigrahi et al., 2020). However, complete surgical removal of a giant tumor is rarely performed due to the technical complexity and the greater risk of side effects. Radio- and chemotherapy have a limited role in the treatment of giant prolactinomas; on the one hand, because of its dubious chemotherapy effectiveness and, on the other hand, because of the complications that appear during tumor irradiation (Iglesias et al., 2018). Therefore, a thorough and deeper understanding of the molecular mechanisms underlying drug resistance of PAs with aggressive behavior like aggressive prolactinomas are urgently needed to find potential new targets for improving therapeutic efficacy. For instance, Jian et al. (2019) demonstrated that miR-145-5p was greatly downregulated in bromocriptine-resistant prolactinoma cell lines and tissues *in vitro* and *in vivo*. In addition, transfer miR-145 mimic into tumor cells and revealed that overexpression of miR-145-5p increased sensitivity for bromocriptine markedly. In conclusion, identification of tumor protein, translationally controlled 1 (*TPT1*) as a direct target gene of miR-145-5p. In another study, miR-93-5p was related to fibrosis and was involved in the bromocriptine -resistance mechanisms in prolactinoma by regulating the transforming growth factor beta 1/mothers against decapentaplegic homolog 3 (*TGF-*β*1*/*Smad3*) signaling pathway (Hu et al., 2019). Interesting that previous studies showed that TGF-β1 promotes the synthesis and secretion of collagen fibers in fibroblasts and that the TGF-β1/Smad3 signaling pathway is involved in the drug-resistance mechanism of prolactinoma by increasing fibrosis through interactions with fibroblasts (Hu et al., 2018).

### High-Grade Meningiomas

Meningiomas are common tumors of the CNS, originating from the meninges of the brain or spinal cord. Most meningiomas are benign tumors characterized by slow growth and are histologically WHO Grade 1 (Schiff and Alyahya, 2020). However, high-grade meningiomas [atypical (WHO Grade 2) and anaplastic (WHO Grade 3)] exhibit more aggressive biological behavior and are clinically associated with a high risk of recurrence and a less favorable prognosis (Maier et al., 2020). Atypical and anaplastic meningiomas account for about 30% of the total number of intracranial meningiomas (Maier et al., 2020). Atypical and anaplastic meningiomas are often accompanied by invasive growth into the surrounding anatomical structures, which is the main factor limiting the radical nature of the surgery and increasing the frequency of recurrence. The problem of diagnosis and treatment, including resistance to radio- and chemotherapy, of patients with high-grade meningiomas, is still far from its final solution, which cannot but affect the long-term results and the level of mortality and mortality (Zhao L. et al., 2020). A better understanding of the molecular mechanisms involved in meningioma oncogenesis may lead to the identification of new therapeutic targets responsible for therapeutic resistance. Numerous studies have identified multiple signaling pathways involved in the therapeutic resistance of high-grade meningiomas and have suggested many important molecular targets for the development of new drugs for the treatment of high-grade meningiomas that are resistant to radio- and chemotherapy. In particular, growth factors, such as *PDGF*, epidermal growth factor (*EGF*) and their receptors, and cytokines, such as *TGF-*β, serve as major factors in high-grade meningiomas leading to therapeutic resistance (Birzu et al., 2020; Shao et al., 2020).

Unfortunately, studies on the role of miRNAs in the mechanisms of resistance to therapy in meningiomas are limited. For instance, microarray analysis, using the atypical meningioma tissue samples of 55 patients (43 from the radiosensitive and 12 from the radioresistant group), indicated that 14 miRNAs were significantly dysregulated in tumor tissue (Zhang X. et al., 2020). Among them 7 significantly upregulated miRNAs (miR-4286, miR-4695-5p, miR-6732-5p, miR-6855-5p, miR-7977, miR-6765-3p, miR-6787-5p) and 7 significantly downregulated miRNAs (miR-1275, miR-30c-1-3p, miR-4449, miR-4539, miR-4684-3p, miR-6129, miR-6891-5p) in patients resistant to radiotherapy. Furthermore, in order to investigate the signaling pathways affected by the differentially expressed 14 miRNAs between radiosensitive and radioresistant atypical meningioma, the authors used the DIANA-miRPath software and found three enriched pathways: two pathways were fatty acid biosynthesis and metabolism, and *TGF-*β signaling pathway. The role of *TGF-*β and these miRNAs in the oncogenesis, particularly radiosensitivity, of meningiomas remains to be established.

## Extracellular Micrornas in Tumor Resistance

**FIGURE 3:**
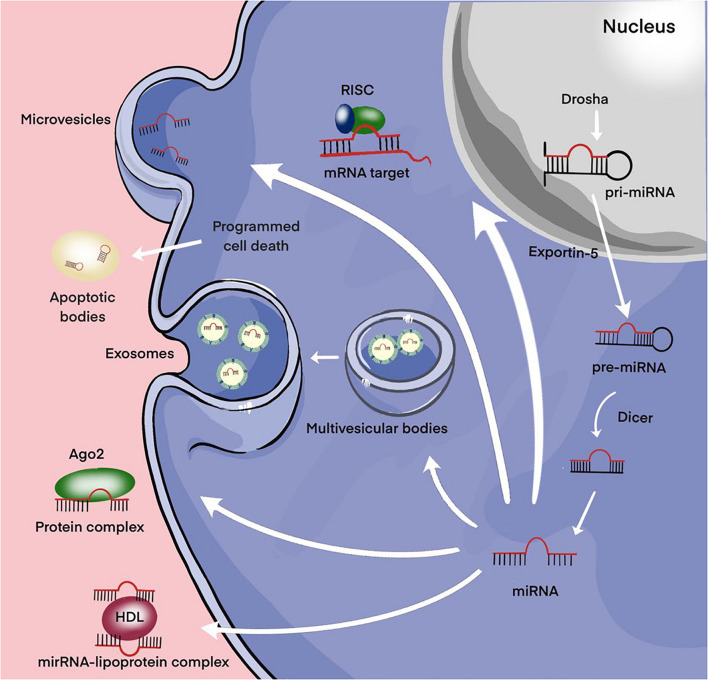
Secretion of miRNAs into the extracellular environment. Primary microRNA (pri-miRNAs) are transcribed by RNA polymerase II and then processed by Drosha into precursor-miRNAs (pre-miRNAs). Exportin5 transfers these pre-miRNAs from the nucleus to the cytoplasm, where Dicer converts them into mature miRNAs. Mature miRNAs can be selectively incorporated into extracellular vesicles (EVs) (exosomes and microvesicles) or linked to the Argonaute 2 (Ago2) protein and released into the extracellular environment. Alternatively, they can be attached to high-density lipoprotein (HDL) or contained in apoptotic bodies and then released into the extracellular environment.

The main part of miRNAs is localized inside the cell. However, a certain proportion of miRNAs are present outside the cells and they are called extracellular or circulating miRNAs. A series of studies are devoted to the detection of extracellular miRNAs in various human fluids including whole blood, plasma/serum, saliva, urine, cerebrospinal fluid (Valihrach et al., 2020). In these biological fluids, the total concentration of miRNAs and their ratio varies considerably, which may be due to the peculiarities of the pathological or physiological status of the organism. The discovery of significant changes in the expression level of extracellular miRNAs in various diseases promoted the positioning of these molecules as promising non-invasive biomarkers (Sohel, 2020). MiRNAs can be secreted by the cell as part of extracellular vesicles (EVs) (exosomes and microvesicles) or apoptotic bodies; they can be found in the form of high-density lipoprotein (HDL) bound and mostly in the form of Argonaute2-containing ribonucleoprotein complexes (miRNA-Ago2) (Figure 3; Valihrach et al., 2020). Then, regardless of the forms, miRNAs pass from the extracellular space into the biological fluid (for example, the general blood flow).

**FIGURE 4:**
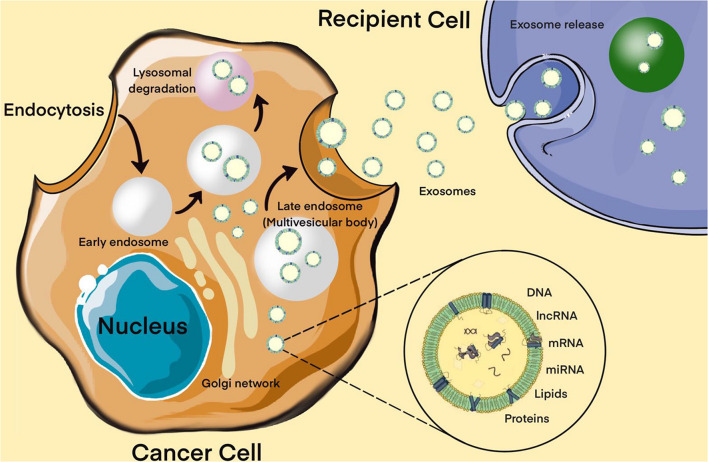
Biogenesis of exosomes. The exosome membrane is formed as a result of the invagination of the early endosome into the membrane. Proteins, lipids, RNA, DNA enter the exosome from the cell cytoplasm. The fate of an endosome depends on the marking of its membrane with certain lipids: if it is labeled with lysobisphosphatidic acid, then its contents will be destroyed, and if ceramides, it will be pushed out of the cell. These processes are controlled by the GTPases of the Rab (G-protein) family, whose various members perform different functions: Rab5 directs the formation of the endosome, Rab7 organizes the degradation of the contents of the multivesicular body (late endosome) in the lysosome, and Rab11, Rab27, and Rab35 are necessary for the secretion of exosomes into the extracellular space. It has been shown that exosomes contain about 4,000 different proteins, more than 1,500 different miRNAs and mRNAs, as well as DNA. Bottom right – enlarged “generalized” exosome.

Moreover, extracellular miRNAs, as exosomal miRNAs, play an important role in intracellular communication and the signaling system of cells. Over the past two decades, a broad evidence base has been obtained regarding the role of extracellular miRNAs in maintaining cellular homeostasis. Exosomes are a new form of intercellular communication (Pegtel and Gould, 2019). These are small membrane vesicles of endosomal origin with a diameter of 30 to 100 nm, which are secreted by various types of cells, normal or abnormal (Pegtel and Gould, 2019). Tumor cells play a particularly important role in the production of exosomes (Kalluri and LeBleu, 2020). In addition to miRNAs, exosomes contain a diverse set of molecules such as DNA, proteins, other non-coding RNAs, translation factors, metabolic enzymes, etc. There is evidence that exosomal miRNAs are actively involved in the oncogenesis of MPBTs, including resistance to therapy (Zhang and Yu, 2019). The presence of surface protein markers and adhesion molecules allows exosomes to bind to cells (including tumor cells) that exhibit the corresponding receptors, via micropinocytosis or endocytosis, to be transported into these cells. All this suggests that miRNAs carried by exosomes can enter recipient cells and regulate the expression of their target genes (Figure 4; Pegtel and Gould, 2019; Kalluri and LeBleu, 2020).

### Glioblastoma

If we talk about the role of EVs in the formation of a resistant phenotype of tumor cells, then, of course, one of the most studied areas is the participation of exosomes in the development of multiple drug resistance (MDR) (Zhang and Yu, 2019). Further studies made it possible to significantly expand the list of biomolecules that are part of resistant tumor cell EVs and are capable of inducing MDR in recipient cells. This primarily refers to miRNAs that regulate the expression level of a number of genes. For instance, Munoz et al. demonstrated that anti-miR-9 delivered via exosomes from MSCs to the TMZ-resistant glioblastoma cells was able to reduce the endogenous upregulation of miR-9 in response to TMZ (Koritzinsky et al., 2017). Moreover, to determine whether multidrug resistance gene 1(*MDR1*) expression is miR-9 dependent for sensitivity to TMZ, the authors knocked down *MDR1* with short hairpin RNA (shRNA), and then examined whether this affects the sensitivity of glioblastoma cells to TMZ. The results indicated that anti-miR-9 increased active caspase by decreased the expression of *MDR1* and concomitantly caused enhanced glioblastoma cell death in response to TMZ treatment. At the same time, in the protocol using manumycin A, which prevented the release of vesicles, it was indicated that the transfer of anti-miR-9 occurs by vesicular transfer, particularly via exosomes.

It should be noted that not only miRNAs but also mRNAs could be transported by EVs. Therefore, exosomes secreted by glioblastoma cells are enriched in mRNA, which enzymes of DNA repair as methylation of the O(6)-Methylguanine-DNA methyltransferase (MGMT) promoter and alkylpurine–DNA–N-glycosylase (APNG), and the transfer of these mRNAs into recipient cells can significantly increase the level of chemoresistance (Shao et al., 2015).

While the participation of EVs in the formation of chemoresistance of tumor cells is not in doubt today, the role of EVs in the regulation of the response of cells to irradiation has been studied to a much lesser extent. Only a few studies are known in which the participation of EVs in the development of the tumor response to radiation has been demonstrated, and the results of these studies are rather contradictory. This is primarily due to the bystander effect, a well-known biological process, radio-induced changes transmitted from irradiated cells to non-irradiated ones (Li and Nabet, 2019). It is assumed that this effect is based on the transmission of induced or radiation-modified biometabolites (reactive oxygen species, cytokines, growth factors, nucleic acid fragments, etc.) non-irradiated cells either by paracrine pathway or through intercellular gap junctions (Ni et al., 2019). In recent years, studies have appeared in which, in on both normal and tumor cells, direct evidence has been obtained for the participation of EVs of irradiated cells in the induction of radiation changes (primarily, genetic instability, telomere contraction) in non-irradiated cells. At the same time, EVs of irradiated cells can also have protective properties, in particular, in experiments on glioblastoma cells, the ability of exosomes from irradiated cells has been described to promotes a migratory phenotype of non-irradiated cells, as the authors believe, as a result of the active accumulation of DNA repair enzymes in the exosomes of irradiated cells (Arscott et al., 2013).

### Pituitary Adenomas With Aggressive Behavior

Hormone therapy is one of the most common types of treatment for hormone-dependent malignant neoplasms, primarily PAs with aggressive behavior. Hormone therapy is based on the principle of creating an artificial deficiency of hormones necessary for the growth of hormone-dependent tumors, which is achieved mainly in two ways: (1) by reducing the concentration of endogenous hormones and (2) by suppressing their synthesis or replacing hormones with their inactive analogs (Iglesias et al., 2020; van Bunderen and Olsson, 2021). Despite the unconditional effectiveness, the use of hormonal therapy is limited by the development of tumor resistance to hormones. The mechanism of hormonal resistance is well understood. Much less is known about the role of intercellular interactions in the development of hormonal resistance of tumors and, in particular, PAs. For instance, Zhao et al. (2021) demonstrated that 20 differentially expressed miRNAs were identified in human invasive and non-invasive PAs tissue, and rat PA cells, where among them, the expression level of miR-99a-3p and mir-149 was significantly reduced. Furthermore, it was shown that overexpression of miR-149 and miR-99a-3p inhibits the growth and metastasis of PA cells and the formation of EC tubes. Interestingly, delivery of miR-149 mimic and miR-99a-3p mimic via exosomes showed similar suppressive effects on cell viability, metastasis, tube formation ability, tumor growth *in vivo*, and expression of markers associated with angiogenesis as *VEGF*. In additional, the authors showed that *NOVA1*, denticleless E3 ubiquitin protein ligase homolog (*DTL*), and *RAB27B* were targeted by miR-99a-3p. It is known that this group of genes is directly promoted EMT in various human tumors (Aleksakhina et al., 2019). Finally, EMT has been shown to contribute to drug resistance in tumors (Du and Shim, 2016; Aleksakhina et al., 2019). Therefore, we can suggest that these miRNAs can be involved in the oncogenesis of PAs, and in particular, be responsible for drug resistance through intercellular communications. In another pilot study to discover that the expression levels of circulating miR-200a in plasma of patients with invasive PAs were significantly higher than that in plasma of patients with non-invasive PAs. Moreover, invasive PA patients with residual after surgery had lower expression levels of circulating miR-200a. Therefore, miR-200 was a potential influencing factor for invasiveness in PAs patients. However, further research is required for exploring the relationships between circulating miR-200a expression and tumor size, clinical characteristics, and molecular mechanism for packaging and secretion to biofluids of miR-200a (Beylerli et al., 2021).

### High-Grade Meningiomas and Medulloblastoma

Aberrant expression of extracellular miRNA circulating in biofluids of certain brain tumor patients has recently been reported to be non-invasive biomarkers and potential regulators of the disease (Sohel, 2020; Valihrach et al., 2020). However, the existence and role of miRNAs in MB and malignant meningioma extracellular environment are unknown. Therefore, better understanding of extracellular miRNA secretion and function in MB and malignant meningioma seems crucial for the development of novel insights for its regulation of oncogenesis including resistance to therapy. For instance, Choi et al. (2020) investigated whether secreted exosomal miR-135b and miR-135a function at the microenvironment level by possibly impacting the stemness of brain tumor spheroid-forming cells (BTSCs). The authors suggest that the inhibition of miR-135b or miR-135a can suppress the self-renewal capacity and expression of stem cell-related markers of BTSCs. In additional, they demonstrated that miR-135b, miR-135a targeted angiomotin-like2 (*AMOTL2*), and the expression of *AMOTL2* can be increased through miR-135b and miR-135a inhibition. This result might be a clue that exosomal miR-135b and miR-135a derived from BTSCs may be able to regulate the Hippo pathway via *AMOTL2*, which plays a significant role in chemoresistance (Mohajan et al., 2021; Zeng and Dong, 2021).

Negroni et al. (2020) using reverse transcriptase real-time quantitative polymerase chain reaction (qRT-PCR) assay demonstrated that exosomal miR-497 and miR-195 are downregulated in serum of patients with high-grade meningioma (WHO grade 2 and 3) compared to benign meningioma (WHO grade 1). However, receiver operating characteristic (ROC) curve analysis showed that exosomal miR-497 has better sensitivity and specificity than exosomal miR-195 in distinguishing between low-grade (WHO grade 1) and higher-grade (WHO grade 2 and 3) meningioma patients, where area under the curve (AUC) was 0.89 and 0.78, respectively. Furthermore, the authors demonstrated that the transcription factor GATA binding protein 4 (GATA-4) is overexpressed in malignant meningioma, which in turn regulates Cyclin D1, and it negatively regulates the miR-497 expression with an increase in cell viability *in vitro.* Importantly, that a cell cycle protein cyclin D1 is an established cancer-driving protein (Montalto and De Amicis, 2020). In recent years, studies have reported that the high expression of Cyclin D1 is involved in drug resistance processes such as chemo- and radiation treatment, and targeted therapy in various human tumors (Liu et al., 2020; Zuo et al., 2021). Therefore, the clinical values of exosomal miR-497 in the therapy resistance of high-grade meningioma must investigate in the future.

### Discussion and Implications

The introduction of extracellular miRNAs into clinical practice is quite active. The search results of the clinical trial database clinicaltrials.gov for the keywords “extracellular,” “vesicle,” “exosomes,” “miRNA,” and “tumor” contain more than 50 projects, some of which are in the recruitment phase. A number of projects have been launched to assess the effectiveness of tumor therapy. In previous studies, it was shown that metformin has cytotoxicity and decreases the viability of glioblastoma cells, a promising biguanide with pronounced antitumor activity (Al Hassan et al., 2018). Furthermore, Soraya et al. (2021) demonstrated that metformin significantly decreased the expression of miR-21, miR-155, and miR-182, indicating suppression of oncogenesis in glioblastoma cells. In addition, confirm metformin increased the exosome biogenesis and secretion in glioblastoma cells. Their result also showed that the expression level of Rab27a, Rab27b, and Rab11 upregulated in treated cells with metformin than that of control cells. In the author’s opinion, the decreased expression levels of miR-21 and miR-182 may be responsible for Rab genes upregulation, which may correlate with increased exosomes secretion.

It turned out that not only exosomes of resistant cells, but also exosomes produced by cells of the tumor stroma can induce drug resistance in tumor cells. In experiments on head and neck cancer, it was found that exosomes produced by tumor-associated fibroblasts are able to induce cisplatin resistance in nearby tumor cells by transferring miR-196a and through targeting cyclin-dependent kinase inhibitor 1B (*CDKN1B*) and inhibitor of growth protein 5 (*ING5*) (Qin et al., 2019).

Another, no less important question is to what extent EVs can affect the initial level of tumor radiosensitivity and, given their protective properties, contribute to the spread of radioresistance to the brain tumors population. Research in this direction is just beginning, and we can expect that soon it will be possible to get answers to this and other questions concerning the role of EVs in the tumor response to radiation.

Thus, in recent years, extensive information has been accumulated on the correlations of miRNA profiles of EV s and the development of a resistant phenotype of tumor cells. Despite significant advances in research on the role of extracellular miRNAs in therapeutic resistance in MPBTs, studies besides glioblastoma with other tumors remains to be seen. In conclusion, the most common miRNAs in EVs that have been reported to be involved in glioblastoma therapeutic resistance are shown in Table 4 (Zeng et al., 2018a).

**TABLE 4:** MiRNAs in extracellular vesicles (EVs) and their signaling pathways through which they may be responsible for glioblastoma therapeutic resistance.

miRNAs	Drug/radiation	Vehicle	Signaling pathways	Regulation	Effect	References
miR-151a	TMZ	Exosome	*XRCC4*	Up	Development of acquired resistance to TMZ	Zeng et al., 2018a
miR-301a	Ionizing radiation	Exosome	*Wnt/b-catenin/TCEAL7*	Up	Depresses radiation sensitivity	Yue et al., 2019
miR-221	TMZ	Exosome	*DNM3*	Down	Inhibit cell proliferation, migration, and TMZ resistance	Yang J. K. et al., 2017
miR-34a	TMZ	Exosome	*MYCN*	Up	Inhibit cell proliferation, migration, invasion and TMZ resistance	Wang B. et al., 2019
miR-124	TMZ	Exosome	*CDK6*	Up	Development of acquired resistance to TMZ and decreases the migration of tumor cells	Sharif et al., 2018
miR-603	Ionizing radiation	EV	*IGF1, IGF1R*, and *MGMT*	Up	Promotes the CSC state and up-regulated DNA repair to promote acquired resistance. Therapeutic platforms hold translational potential in the treatment of wtIDH/umMGMT glioblastoma	Ramakrishnan et al., 2020
miR-93 and miR-193	TMZ	Exosome	*Cyclin D1*	Up	Decreases cell cycling quiescence and contribute to TMZ resistance	Munoz et al., 2019
miR-1238	TMZ	Exosome	*CAV1/EGFR*	Down	Inhibit to TMZ resistance	Yin et al., 2019
miR-21-5p	Pacritinib + TMZ	Exosome	*STAT3/PDCD4*	Down	Inhibit to drug resistance	Chuang et al., 2019
miR-27a-3p, miR-22-3p and miR-221-3p	Ionizing radiation	EV	*CHD7*	Up	Promote PMT in GSCs. Inhibit radioresistance	Zhang Z. et al., 2020

**XRCC4*, X-ray repair cross-complementing protein 4; *TCEAL7*, transcription elongation factor A protein-like 7; *DNM3*, dynamin 3; *CDK6*, cyclin-dependent kinase 6; *IGF1*, insulin-like growth factor 1; *IGF1R*, insulin-like growth factor 1 receptor; *MGMT*, O(6)-Methylguanine-DNA methyltransferase; *CAV1*, caveolin 1; *EGFR*, epidermal growth factor receptor; *STAT3*, signal transducer and activator of transcription 3; *PDCD4*, programmed cell death protein 4; *CHD7*, chromodomain-helicase-DNA-binding protein 7; miR, microRNA; TMZ, Temozolomide; PMT, proneural-to-mesenchymal transition; GSCs, glioma stem cells.*

## Conclusion

The last decade has been accompanied by the emergence of a large number of studies devoted to the role of miRNAs in oncogenesis and the development of resistance to antitumor therapy. The discovery of EVs and, most importantly, their ability to transfer biological material as miRNAs from cell to cell has largely changed the understanding of the mechanism of tumor development and progression. First, this is the revealed ability of EVs to induce tumor transformation and/or induce a tumor-like phenotype in the cells of the surrounding tissue. Moreover, of course, one of the most significant achievements in this reign – the influence of EVs on the formation of a resistant phenotype of tumor cells. EVs can indeed provide the spread of resistance from resistant to sensitive cells through various mechanisms based on the transfer of specific regulatory molecules into cells including proteins, miRNAs, mRNA, etc. The induction of resistance to chemoradiotherapy and pharmacological treatment of MPBTs by miRNAs has been convincingly demonstrated in *in vitro* and *in vivo* experiments. However, there are a number of questions. And one of them in which To the extent that miRNAs are involved in the development of tumor resistance to antitumor therapy, can miRNAs actually participate in the development of resistance across the entire pool of tumor cells under *in vivo* conditions, and, most importantly, how important this process is in the development of acquired tumor resistance. Today these issues are being actively investigated, and, of course, their solution will make it possible to make significant progress in solving such an important problem in neurosurgery as the resistance of MPBTs to antitumor therapy.

## Author Contributions

IG: conceptualization, writing – original draft, and supervision. OB: writing – review and editing, investigation, project administration, and resources. AA: formal analysis, methodology, and original draft. YL, HX, CL, XX, and CY: data curation. GY: validation, visualization, and funding acquisition. All authors have read and agreed to the published version of the manuscript.

## Conflict of Interest

The authors declare that the research was conducted in the absence of any commercial or financial relationships that could be construed as a potential conflict of interest.

## Publisher’s Note

All claims expressed in this article are solely those of the authors and do not necessarily represent those of their affiliated organizations, or those of the publisher, the editors and the reviewers. Any product that may be evaluated in this article, or claim that may be made by its manufacturer, is not guaranteed or endorsed by the publisher.
